# Supplementary material for: Respiratory and Otolaryngology Symptoms Following the 2019 Spring Floods in Quebec
Source: Int J Environ Res Public Health. 2022 Sep 17;19(18):11738. doi: 10.3390/ijerph191811738 (PMC9517661; doi:10.3390/ijerph191811738)
Supplement: Supplementary file 1 [file ijerph-19-11738-s001.zip › ijerph-1878376-supplementary.pdf]

Supplementary Materials

Questionnaire

[REGION] In which region do you live?

[r1] Laurentides

[r2] Laval

[r3] Mauricie-Centre du Québec

[r4] Montérégie

[r5] Montréal

[r6] Outaouais

[r7] Other region

[REGION2] Do you live in Sainte-Marthe-sur-le-Lac, or in another municipality in the Laurentides?

[r1] Sainte-Marthe-sur-le-Lac

[r2] Other municipality

[Q01] What is the language you first learned at home in your childhood and still understand?

[r1] French

[r2] English

[r3] Other

[r4] French and English

[r5] French and other

[r6] English and other

[Q1] Is this the telephone number of your main residence, which could either be a dwelling unit or a private house?

[r1] Yes

[r2] No

[r99] I don't know/I prefer not to answer

[Q2] Do you live in a collective household, such as a rooming house, nursing home, student or senior residence?

[r1] Yes

[r2] No

[r99] I don't know/I prefer not to answer

[Q3] Do you (or does a member of your household) own your home?

[r1] Yes

[r2] No

[r99] I don't know/I prefer not to answer

[Q4] Is your home located in a:

Row:

[r1] Detached single-family home

[r2] Townhouse or semi-detached house

[r3] Duplex or triplex

[r4] Building (4 or more floors)

[r96] Other, specify:

[r99] I don't know/I prefer not to answer

[Q5] Could you give us the six-character postal code of the main residence where you currently live?

[Q6] Was the place where you were living during the floods of spring 2019 located at the same postal code?

[r1] Yes

[r2] No

[r99] I don't know/I prefer not to answer

[Q6B] In which postal code was this residence or dwelling unit located?

[Q7] In which municipality was your main residence located during the 2019 spring floods?

[r96] Please enter the municipality's name.

[r99] I don't know/I prefer not to answer

[sexe] [pipe: SEXTXT]

Note: As indicated by Statistics Canada, transgender, transsexual, and intersex Canadians should indicate the sex (male or female) with which they most associate themselves.

[r1] Male

[r2] Female

[age] How old are you?

[r0] Under 18

[r1] Between 18 and 24

[r2] Between 25 and 34

[r3] Between 35 and 44

[r4] Between 45 and 54

[r5] Between 55 and 64

[r6] Between 65 and 74

[r7] 75 or older

[r9] I prefer not to answer

[Q9] In what year were you born?

[Q10] What is your current marital status?

[r1] Married and not separated

[r2] Common-law union

[r4] Separated

[r5] Divorced

[r6] Widow/widower

[r3] Single (never legally married)

[r99] I don’t know/I prefer not to answer

[Q11] Was your marital status the same during the 2019 spring floods?

[r1] The same

[r2] Different

[r99] I don’t know/I prefer not to answer

[Q11B] Please specify your marital status:

[r1] Married and not separated

[r2] Common-law union

[r4] Separated

[r5] Divorced

[r6] Widow/widower

[r3] Single (never legally married)

[r99] I don’t know/I prefer not to answer

[Q12] Currently, excluding you, how many people usually live with you at least half the time?

[r97] Zero

[r1] One

[r2] Two

[r3] Three

[r4] Four

[r5] Five or more

[r99] I don’t know/I prefer not to answer

[Q13] Excluding you, how many people usually lived with you at least half the time during the 2019 spring 2019 floods?

[r97] Zero

[r1] One

[r2] Two

[r3] Three

[r4] Four

[r5] Five or more

[r99] I don’t know/I prefer not to answer

[Q14] During the 2019 spring floods, among the people who usually lived with you at least half the time, how many were:

[r1] Under 2 years of age:

[r2] 2 to 17 years of age:

[r3] 18 to 64 years of age:

[r4] More than 64 years of age:

[Q17] Compared to other people of the same age, would you say your health is:

[r1] Excellent

[r2] Very good

[r3] Good

[r4] Fair

[r5] Bad

[r99] I don’t know/I prefer not to answer

[Q18] Since the 2019 spring floods, would you say that your health:

[r1] Has remained stable

[r2] Has improved

[r3] Has declined

[r99] I don’t know/I prefer not to answer

[Q19] In connection with the 2019 spring floods, were you or was anyone living in your home physically injured (cut, burn, sprain, etc.)?

[r1] Yes

[r2] No

[r99] I don’t know/I prefer not to answer

[Q20] How often do you currently smoke cigarettes (including cigars, cigarillos or pipes)?

[r1] Every day

[r2] Occasionally

[r3] Never

[r99] I don’t know/I prefer not to answer

[Q21] In the past six months, how many times have you had five or more drinks of alcohol on one occasion?

Note: By a “drink,” we mean a beer, a glass of wine, a glass or cocktail containing an ounce and a half of spirits or strong liquor.

[r1] Never

[r2] Less than once a month

[r3] Once a month

[r4] Two or three times a month

[r5] Once a week

[r6] More than once a week

[r99] I don’t know/I prefer not to answer

[Q22] Since the 2019 spring floods, would you say that your alcohol consumption…?

[r1] Has increased

[r2] Has remained stable

[r3] Has declined

[r99] I don’t know/I prefer not to answer

[Q23] Have you ever been diagnosed by a doctor with any of the following diseases?

Column:

[c1] Yes

[c2] No

Row:

[r1] Asthma

[r2] Rhinitis (for example: allergic, hay fever...)

[r3] Eczema or hives

[r4] Chronic bronchitis

[r5] Emphysema

[r6] Other respiratory disease (which one)

[Q24] Are you currently taking any medications for the following diseases?

[r1] Asthma

[r2] Rhinitis (for example: allergic, hay fever...)

[r3] No medications for these diseases

[r99] I don’t know/I prefer not to answer

[Q25] In the past six months, how often have you had the following symptoms (other than the flu, colds or seasonal allergies)?

Column:

[c1] Once a week or less

[c2] 2 or 3 times a week

[c3] Almost every day

Row:

[r1] Headaches

[r2] Unusual fatigue

[r3] Watery eyes

[r4] Red eyes that burn and/or sting

[r5] Stuffy nose

[r6] Runny nose

[r7] Sneezing

[r8] Dry nose

[r9] Nose bleed

[r10] Pressure or pain in the ear

[r11] Sore throat

[r12] Dry throat

[r13] Secretions in the throat

[r14] Cough

[r15] Sputum

[r16] Wheezing

[r17] Shortness of breath

[r18] Chest tightness

[r19] Redness of the skin

[r20] Itching

[Q26] At this time, for each of the following symptoms, would you say this symptom...

Column:

[c1] Don’t change

[c2] Is worse outside of your home

[c3] Is better outside of your home

Row:

[r1] Headaches

[r2] Unusual fatigue

[r3] Watery eyes

[r4] Red eyes that burn and/or sting

[r5] Stuffy nose

[r6] Runny nose

[r7] Sneezing

[r8] Dry nose

[r9] Nose bleed

[r10] Pressure or pain in the ear

[r11] Sore throat

[r12] Dry throat

[r13] Secretions in the throat

[r14] Cough

[r15] Sputum

[r16] Wheezing

[r17] Shortness of breath

[r18] Chest tightness

[r19] Redness of the skin

[r20] Itching

[Q27] Do you have access to a family doctor?

[r1] Yes

[r2] No

[r99] I don’t know/I prefer not to answer

[Q28] For the past six months, would you say your mental health is:

[r1] Excellent

[r2] Very good

[r3] Good

[r4] Fair

[r5] Bad

[r99] I don’t know/I prefer not to answer

[Q29] Do you have a mood disorder, such as depression, bipolar disorder, mania or dysthymia?

[r1] Yes

[r2] No

[r99] I don’t know/I prefer not to answer

[Q30] Do you have an anxiety disorder, such as a phobia, obsessive-compulsive disorder or panic disorder?

[r1] Yes

[r2] No

[r99] I don’t know/I prefer not to answer

[Q31] In the past six months, have you used sedatives or tranquilizers?

[r1] Yes

[r2] No

[r99] I don’t know/I prefer not to answer

[Q32] In the past six months, have you used antidepressants?

[r1] Yes

[r2] No

[r99] I don’t know/I prefer not to answer

[Q33] Over the past month, how often have you felt…

Column:

[c0] Never

[c1] Rarely

[c2] Sometimes

[c3] Most of the time

[c4] Always

Row:

[r1] Nervous

[r2] Desperate

[r3] Restless or not in place

[r4] So depressed that nothing could make you smile

[r5] That everything was an effort

[r6] Good for nothing

[Q34] Thinking about the level of stress in your life, would you say that most of your days are:

[r1] Not stressful at all

[r2] Not that stressful.

[r3] A bit stressful

[r4] Rather stressful

[r5] Extremely stressful

[r99] I don’t know/I prefer not to answer

[Q35] What is your main source of stress?

[r1] Work

[r2] Financial worries

[r3] Family

[r4] Lack of time

[r5] Personal problems and other

[r6] Health

[r7] School

[r97] No source of stress

[r96] Other, specify:

[r99] I don’t know/I prefer not to answer

[Q36] For each of the proposed comments that conern the 2019 floods, please indicate how these comments applied to you during the LAST SEVEN DAYS. If some comments did not apply to you, please answer “not at all.”

Column:

[c1] Not at all

[c2] Rarely

[c3] Sometimes

[c4] Often

Row:

[r1] I thought about it when I did not mean to.

[r2] I avoided letting myself get upset when I though about it or was reminded about it.

[r3] I tried to remove it from my memory.

[r4] I had trouble falling asleep or staying asleep because of pictures or thoughts about it that came to my mind.

[r5] I had waves of strong feelings about it.

[r6] I had dreams about it.

[r7] I stayed away from reminders about it.

[r8] I had the impression that it had not happened or was not real.

[r9] I tried not to talk about it.

[r10] Pictures about it popped into my mind.

[r11] Other things kept making me think about it.

[r12] I was aware that I still had a lot of feelings about it, but I didn't deal with them.

[r13] I tried not to think about it.

[r14] Any reminder brought back feelings about it.

[r15] My feelings about it were kind of numb.

[Q37] During the floods, did you have any difficulty getting access to the following services by car or public transit?

Column:

[c1] Yes

[c2] No

[c3] Not applicable

Row:

[r1] Work

[r2] Stores/shopping centre

[r3] Health care

[r4] Social services

[r5] Social activity

[r6] Day care, school

[Q37B] For how many days were the following services interrupted at home?

[r1] Work

[r2] Stores/shopping centre

[r3] Health care services

[r4] Social services

[r5] Social activity

[r6] Day care, school

[Q38] During the 2019 spring floods, was your yard flooded?

[r1] Yes

[r2] No

[r99] I don't know/I prefer not to answer

[Q39] During the 2019 spring floods, was your home flooded?

[r1] Yes

[r2] No

[r99] I don't know/I prefer not to answer

[Q40] During the 2019 spring floods, was at least one habitable room in your home flooded?

Note: By habitable room, we mean: a room that you occupy for at least three months a year, such as the living room, bedroom, kitchen, or dining room. It does not include any corridors or the garage.

[r1] Yes, at least one habitable room was flooded

[r97] No

[r2] Non, mais des pièces non habitables ont été inondées

[r3] Non, aucune pièce inondée, mais ma rue a été inondée

[r4] Non, rien de cela

[r99] I don’t know/I prefer not to answer

[Q41] When the inside of your house was flooded, what was the water level (from the ground floor)?

[r1] Less than 1 foot/30 cm

[r2] 1 to 3 feet/30 to 100cm

[r3] More than 3 feet/100cm

[r99] I don’t know/I prefer not to answer

[r97] Not applicable

[Q42] What is your assessment of the extent of the damage to the following items during the 2019 spring floods?

Column:

[c1] Total loss

[c2] Major damage

[c3] Minor damage

[c4] No damage

Row:

[r1] House

[r2] Yard

[r3] Furniture and electronic equipment

[r4] Contents of the garage or shed

[r5] Vehicle

[r6] Tools

[r7] Office equipment and books

[r8] Clothing

[r9] Toys

[r10] Recreational equipment

[r11] Items of sentimental value (photos, souvenirs...)

[Q43] What is your estimate of the total cost of your material losses related to the 2019 spring floods?

[r1] No loss

[r2] Less than $5000

[r3] Between $5000 and $24,999

[r4] Between $25,000 and $49,999

[r5] Between $50,000 and $99,999

[r6] More than $100,000

[r99] I don’t know/I prefer not to answer

[Q44] Who cleaned and repaired the rooms affected by the flood?

[r1] I did (by myself)

[r2] I did, with the help of members of my immediate family (spouse, children)

[r3] I did, with the help of my relatives

[r4] I did, with the help of friends or neighbours

[r5] Friends, neighbours, relatives of mine without my being able to help them

[r6] An independent worker (self-employed)

[r7] Volunteers from a community organization

[r8] A cleaning company

[r9] A construction company

[r96] Other, specify:

[r97] I did not do any cleaning or repair work

[Q45] Did you have to leave your home because of the 2019 spring floods?

[r1] Yes

[r2] No

[r99] I don’t know/I prefer not to answer

[Q46] For how long?

[r1] Less than a week

[r2] 1 to 2 week(s)

[r3] 3 to 4 weeks

[r4] More than a month

[Q47] When you left your home, who took you in?

Row:

[r1] Accommodation centre for disaster victims (under the supervision of the Red Cross)

[r2] Family

[r3] Friends

[r4] Neighbours

[r5] Hotel/motel

[r6] Chalet or caravan (secondary residence)

[r96] Other, specify:

[r99] I don’t know/I prefer not to answer

[Q48] Currently, can you reuse all habitable parts of your home in a normal fashion, even those that were damaged in the spring of 2019?

Row:

[r1] Yes

[r2] No

[r99] I don’t know/I prefer not to answer

[Q49] During the floods, were any of the following services interrupted at home? If so, for how many days?

Column:

[c1] Yes

[c2] No

[c3] Not applicable

Row:

[r1] Drinking water

[r2] Electricity

[r3] Draining system

[r4] Mail delivery

[r5] Phone line

[r6] Internet

[r7] Home-based social services (e.g., social worker)

[r8] Home care services

[Q50] For how many days were the following services interrupted at home?

[r1] Drinking water

[r2] Electricity

[r3] Draining system

[r4] Mail delivery

[r5] Phone line

[r6] Internet

[r7] Home-based social services

[r8] Home care services

[Q51] Were you insured for the 2019 spring floods?

[r1] Yes

[r2] No

[r99] Je ne sais pas/Je préfère ne pas répondre

[r8] I don’t know

[r9] I prefer not to answer

[Q52] Have you submitted an application to the Quebec government’s program for flood victims for the 2019 floods?

[r1] Yes

[r2] No

[r99] Je ne sais pas/Je préfère ne pas répondre

[r8] I don’t know

[r9] I prefer not to answer

[Q53] Have you received funds or donations from any of the following sources to cover your damages?

Column:

[c1] Yes

[c2] No

[c3] Not applicable

Row:

[r4] Donations from family or friends

[r1] Insurance

[r2] Government

[r3] Red Cross

[Q54] Following the losses and damages you incurred after the 2019 spring floods, do you consider that the amounts received to pay for the expenses cover:

[r1] All or most of the costs incurred

[r2] About half of the costs incurred

[r3] Less than half of the costs incurred

[r4] You have not received any financial assistance

[r99] I don’t know/I prefer not to answer

[Q55] Have you had to take out bank loans or any other type of loans to deal with repairs to your home or material losses as a result of the 2019 spring floods?

[r1] Yes

[r2] No

[r99] I don’t know/I prefer not to answer

[Q56] Concerning the tangible help or moral support to cope with the different stressors you experienced following the 2019 spring floods, would you say you received:

[r1] More help than expected

[r2] As much help as you expected

[r3] Less help than expected

[r99] I don’t know/I prefer not to answer

[Q57] Before the spring of 2019, did you experience any other flooding at home or on your property?

[r1] Yes

[r2] No

[r99] I don’t know/I prefer not to answer

[Q58] Before the spring of 2019, when was your last flood experience?

[Q59] Were you born in Canada?

[r1] Yes

[r2] No

[r99] I don't know/I prefer not to answer

[Q60] If you were not born in Canada, are you a...?

[r1] Canadian citizen

[r2] Landed immigrant (permanent resident)

[r3] Person claiming refugee status (asylum seeker) in Canada

[r4] A temporary resident (work permit, study)

[r99] I don't know/I prefer not to answer

[Q61] What is the highest level of education you have completed?

[r1] Primary

[r2] Secondary

[r3] Trade school, commercial or specialized college

[r4] College (Cégep)

[r5] University

[r99] I don't know/I prefer not to answer

[Q62] What was your total household income (spouse) for 2018 before taxes (e.g., employment, social assistance, employment insurance, retirement pension)?

[r1] Less than $19,999

[r2] $20,000 to $29,999

[r3] $30,000 to $49,999

[r4] $50,000 to $69,999

[r5] $70,000 to $79,999

[r6] $80,000 to $99,999

[r7] $100,000 or more

[r99] I don't know/I prefer not to answer

[Q63] What was your main occupation for the last six months?

[r1] Full-time worker

[r2] Part-time worker

[r3] Self-employed

[r4] Seasonal worker

[r5] Student

[r6] Retired (annuitant)

[r7] Semi-retired

[r8] Homemaker

[r9] Maternity/paternity leave

[r10] Employment Insurance claimant

[r11] Social assistance claimant (income security)/Sick leave / CSST

[r12] Disability/SAAQ

[r96] Other, specify:

[r99] I don’t know/I prefer not to answer
